# Progress in State Medicine

**Published:** 1902-08-23

**Authors:** 


### Progress in State Medicine.

**Cancer.**—Mason[1] collected from the death returns of the registrar upwards of 400 cases of cancer occurring in the Leamington district, extending over a period of 15 years. From analysis of these he states that the alimentary system accounts for 55½ per cent., the glandular for 16 per cent., the generative organs for 13¾ per cent., also that if those cases affecting only the mammary gland and the uterus are deducted, the liability of male and female is about the same. There is no decade in which cancer does not occur; that the greatest liability occurs 10 years earlier, and persists for 10 years longer in the female than in the male. Out of these 427 cases, deaths from cancer occurred in the same house only seven times, and in one of these the patient only moved into that house a few days prior to her death. It is, however, very common for it to occur in houses in the same row and on the same side of the street, and this is probably not accidental or mere coincidence, but that there is some determining cause in common. Of the 427 cases, 44 died in public institutions, previous residences unknown, and deducting seven in which cancer had occurred twice, there remain 376 dwellings in which it occurred. Of these, 66 are end houses of rows, or corner houses of streets; 178 are above the annual rental of £20, and 198 are below that value. There are in Leamington approximately 2,263 houses of the former, and 3,793 of the latter value. This gives a percentage of 7·8 of the better class to 5·2 of the poorer. The distribution of cancer is patchy. In one particular district with a population of 930, where the houses are well built, above the annual rental of £50, and situated on a stiff clay ground, cancer has only occurred in four houses during the 15 years. As regards the nature of the ground upon which the cancer houses abound, he states that 125 were on gravel, 57 sandstone, 3 loose soil, 86 clay, and 105 unascertained. It was only possible to get drainage statistics concerning the last 175 cases. Defective drainage had been reported to the authorities in 45 cases, a percentage of 25·7. Out of these 175 cases, deducting one in which a case had occurred twice, 117 of the houses were upwards of 30 years old, and 57 are less, so that 67 per cent. of these cases occurred in the older houses. These points seem to indicate that, whatever the ultimate cause may be, cancer will be found to be due to some germ whose habitat is a sewage-contaminated subsoil. The most likely mode of entrance of such germ into the organism is through one of the mucous-membrane-lined apertures of the body. Rabagliati [2] refers to the fact that cancer is not yet one of the most important causes of death. Comparing the three causes of mortality, the fevers, consumption, and cancer, they stand to one another, roughly, in the proportion of 4, 2, and 1. The alarming thing about cancer was that it was growing rapidly as a cause of death, and that its victims were people in the prime of life. In the decennium 1861–1870 the mortality in England and Wales was 384 per million; in 1871–80, 468; 1881–90, 589; in 1891–95, 712; and in 1896–99; the last years for which returns were available, the ratio was 795 persons per million per annum. As to the causes of cancer, whatever they were they take time to act, because children were practically exempt from it. Of the 109,376 deaths from cancer in the quinquennium, 1892–96, only 28 per cent. were of children under five years of age. Nearly three-quarters of the total mortality from cancer occurred between the ages of 45 and 75 years. The greater prevalence among women he attributes to their habits of small and frequent meal-taking—meals consisting of cakes, bread, and sweets. There was no evidence in his experience that excessive meat-eating was an important cause of cancer, though there was plenty of evidence that excessive food-consumption was. Owing to an alteration in the habits of men they were becoming more liable to cancer. Thirty-three years ago, for every three men who died from cancer seven women succumbed; now, for every three men only five women. An emphatic contradiction [3] is given to the statement that the Jewish race are less liable to cancer than other races. Mammary scirrhus has often been met with among Jews, and while examples of nearly every form of cancer have been seen, there has seemed to be a special tendency to the development of intestinal malignant growths among them. There is undoubtedly an impression in the minds of the members of the Jewish community that, far from being exempt, the Jews are especially prone to suffer from malignant growths. The returns of the Burial Society of the United Synagogue deal with the deaths occurring in about 85 per cent. of the Jewish community in London. The percentages of deaths from cancer to deaths from all causes of persons over 20 years of age, for the years 1900, 1899, and 1898 are 6·1, 6·5, 5·02 respectively. The Registrar-General's returns for London for the same period for the general population are 8·4, 8·8, 8·9. Taking the Jewish population of London at 80,000, the rate for Jews is 525 deaths from cancer among 1,000,000 persons living, while the rate for England and Wales for 1896 was 764 deaths, and from the Registrar-General's returns for London the rate for 1900 would work out at 800 deaths per 1,000,000 persons living. This discrepancy may be partially accounted for by the fact that considerably more than half the statements as to the cause of death which are made to the officers of the United Synagogue are made verbally, and not accompanied by the medical certificate. A further fallacy underlying these statistics is probably to be found in the age distribution of the Jews in London. For the last twenty years there has been a steady tide of immigration of Jews from Russia and Poland into England. These immigrants chiefly consist of adults from 20 to 30 years of age with their young children. Of these, therefore, only such as arrived at the beginning of the period of immigration would have yet reached the age at which cancer is most likely to occur. The true conclusion seems to be that while cancer is less prevalent among Jews than among the general population, nevertheless the probability that a Jew of cancer age will suffer from that disease is not materially less than in the case of a person of another race. The idea that the consumption of pork may be a cause of cancer is destroyed by the fact that cancer attacks equally the orthodox and heterodox Jews. Banks,[4] whilst of opinion that statistics were very dangerous things to manipulate, says that it is now universally admitted that in all civilised peoples there had been a notable increase in the amount of cancerous disease. His own personal experience of 35 years pointed distinctly to the fact that cancer was beginning to attack younger people. While there was an increase of cancer in both sexes, in the case of the male sex the increase had been greater than in the case of the female. Of the reasons suggested for this increase he could only recognise one prominently, namely, the great increase in the amount of food, and notably of meat, which was now consumed by civilised people. The wretched half-starved patient was not the most prone to cancer; its most numerous victims were well-nourished persons with plenty of fat and often with the colour of health in their cheeks. Opinions varied as to whether there was, or was not, a cancer parasite. He thought there was a special body in cancer, very easily recognisable, when they were able to appreciate it, which at all events accompanied cancer. He did not consider that it was due to a degeneration of the nuclei of the epithelial cells. He deprecated the introduction of fresh theories as to the causation of cancer unless they had been well matured. A census [5] was made on October 15th, 1900, of all patients under treatment for cancer in the country of Holland. Unfortunately medical practitioners did not co-operate as much as they might have done, and the results are therefore to be accepted with caution. The percentage of cases of population was 0·0286, the proportion of males to females was 1 to 1·205. In regard to age the largest number of cases occurred between 61 and 70, next came the periods 51 to 60, and then 71 to 80. In only 3·53 per cent. of the cases was the age of the patient over 80. The situation of the cancer was in 49·88 per cent. the intestinal tract; in 20·20 the breast, including one case in a man; in 12·74 the female genitals; in 7·15 the face; in 3·87 the under lip; in 5·58 per cent. other parts. Of 438 cases of cancer of the intestine, 275 were in men, 163 in women. Among 372 male patients, in 73·9 per cent. the disease affected the digestive organs; whilst in women the proportion was only 32·2 per cent. In 18·1 per cent. of cases cancer had occurred in several members of the family. On the whole hereditary disposition was found in 19·7 per cent. of the cases. The co-existence of cancer in husband and wife was noticed in 11 cases, and the percentage of cases in which infection from person to person might be assumed was 10·92. Previous syphilis was noted in 9 cases, injury in 36, alcoholism in 87, abuse of tobacco in 125. Taking alcoholism and tobacco in relation to the numbers of male patients, the percentages were respectively of 23·4 and 33·7. Payne[6] draws attention to the fact that multiple primary cancer is almost unknown, that is to say that when cancer was once established no new cancer could be acquired. It might be taken for granted that cancer, as a rule, was not a contagious disease. It had not been shown that cancer was not communicable from one person to another as were, for instance, threadworms. In his opinion it was evident that if there was any infective cause of cancer the skin was one of the channels by which it invaded the body. There was another possible channel of invasion, namely, by the internal surface of the body, especially by the alimentary tract. Primary cancer of the lung was almost unknown. It might be said, broadly, that less than 16 per cent. of all cancers began on the external surface of the body. Not less than 56 per cent. of fatal cancers began on the internal surface of the body. In regard to uterine cancer the climateric had to be considered, and it had to be remembered also that any parasite with an accustomed mode of entrance to the body also had an accustomed method of exit. He suggested that mammary cancer was a form of sebaceous cancer, and put forward the speculation that the sebaceous gland represented the excretory path by which cancer germs were eliminated from the body. In uterine and mammary cancer their special localisation was due to abortive elimination of germs, which, if arrested on the outward path, produced a primary cancer. The rate of increase in cancer of the digestive organs was higher than the average for all organs. The most plausible explanation was the more abundant diet of the working classes, and the increased consumption of meat in England; but cancer was increasing in other countries where the economical conditions were not the same. Brand[7] states that his reading and observations had forced him to the belief that the cause of cancer came from without the organism, and that the infection theory was an excellent working hypothesis, and the only one which met the case fully. While primary cancer can be originated in practically any tissue, it has an almost exclusive selective affinity for epithelial surfaces generally, and for the mucous membrane in particular. Wherever cancer grows luxuriantly and rapidly the chief desiderata for the growth of bacteria are found, namely, moisture, suitable medium, constant temperature, and exclusion of light and air. The infectiousness of cancer, like that of leprosy and tubercle, does not seem to be very great. In all probability cancer does not arise in sound and healthy tissues. Irritation or injury renders the tissues less able to withstand the infective agent. Of authentic cases of auto-inoculation of cancer the number is considerable. Ebert collected 23 cases of contact cancer, such as lip to lip, tongue to gum, one labium majus to another, and mentions the case of a woman who inoculated the corner of her eye from a cancer on the back of her hand. Cancer has also been successfully inoculated experimentally, chiefly however on the lower animals. Bose alludes to three cases of inoculation from man to man, done both intentionally and successfully. Brand concludes from these and other experiments that there can no longer be any doubt that cancer is a contagious disease. Geographically, cancer is most rampant where sewage is most difficult to be got rid of, and where it is most likely to be deposited and remain after floodings or high tides on a non-porous soil. This permits of and fosters the prolific growth of micro-organisms; and the frequent occurrence of shallow surface wells in such districts suggests an easy and extensive contamination of drinking water. Referring to cancer houses, and rooms, he mentions a house near London, a room in which was occupied by three women in succession who all died of cancer at fairly short intervals. Each of these women appeared to be in perfect health, when she in turn came to occupy this room, and had lived in other rooms in the same house, but within a year of occupying this room they developed cancer. There was no relationship between them. No further cases of cancer occurred in this house after the room was thoroughly disinfected, and the bedding burnt. With regard to heredity, the children of cancerous parents might possibly acquire more or less vulnerability congenitally, but that it was most improbable that the disease itself could be transmitted. The occurrence of cancer in several members of a family, after the death of parents from that disease, could not be accepted as evidence of heredity; but on the contrary, could and ought to be accepted as evidence of infection from an obvious source. As regards prophylaxis, much might be hoped for from notification, improved sanitation, including disinfection of houses, cremation of bodies, the use of pure drinking water, moderation in diet, abstention from alcohol in excess, and general observance of the known laws of health.

[1] Brit. Med. Jour., Jan. 18, 1902. [2] Sanit. Rec., March 6, 1902. [3] Brit. Med. Jour., March 15, 1902. [4] Lancet, March 22, 1902. [5] Brit. Med. Jour., April 5, 1902. [6] Ibid. [7] Brit. Med. Jour., July 26, 1902.

